# Differential Methylation Analysis of Suicidal Ideation Severity in Schizophrenia with the Illumina MethylationEPIC Array

**DOI:** 10.3390/healthcare10050809

**Published:** 2022-04-27

**Authors:** Kevin Z. Wang, Zanib Chaudhary, Jessica Qian, Christopher Adanty, Ariel Graff-Guerrero, Philip Gerretsen, Clement C. Zai, Vincenzo De Luca

**Affiliations:** Department of Psychiatry, University of Toronto, Toronto, ON M5T 1R8, Canada; kevinz.wang@mail.utoronto.ca (K.Z.W.); zn857242@dal.ca (Z.C.); jessica.qian@camh.ca (J.Q.); christopher.adanty@camh.ca (C.A.); ariel.graff@camh.ca (A.G.-G.); philip.gerretsen@camh.ca (P.G.); clement.zai@camh.ca (C.C.Z.)

**Keywords:** DNA methylation, schizophrenia, suicide, Illumina EPIC, C-SSRS

## Abstract

There is a multitude of factors that makes difficult to identify those at risk for suicide, especially among schizophrenia patients. Suicide cannot be explained by genetics alone, therefore epigenetic mechanisms including DNA methylation are thought to play a role. DNA methylation could be a valuable tool in helping predict those at-risk individuals. This cross-sectional study comprised 112 subjects diagnosed with schizophrenia spectrum disorders, and were grouped according to the current suicidal ideation severity. DNA methylation across the genome was measured with the Infinium^®^ MethylationEPIC BeadChip. We utilized the dmpFinder and bumphunter functions within the Bioconductor minfi package to identify differentially methylated positions (DMPs) and differentially methylated regions (DMRs), respectively. Following quality control, we removed one sample from the analysis and reported the most significant DMPs and DMRs associated with suicidal ideation severity. All positions and regions identified in this analysis were only found to have suggestive levels of significance at the genome-wide level. The present study was one of the first to investigate genome-wide methylation and suicidal ideation severity. While there were many strengths of our study, including investigating both differentially methylated positions and regions, further larger-scale studies are necessary to replicate, support, and validate our findings presented here.

## 1. Introduction

Suicidal behavior has consistently been ranked as one of the leading causes of injury and death worldwide. However, patients with schizophrenia have a tenfold increase in suicide risk compared to that of the general population, with an estimated 25–50% of these individuals making at least one suicide attempt during the course of their lifetime [1,2]. Suicidal ideation greatly increases the risk for later suicide attempts and can serve as an important early warning sign for family, caregivers, and clinicians alike [3,4].

The symptoms of schizophrenia can often complicate the ability to accurately predict patients at high risk for suicide. For instance, positive symptoms such as auditory hallucinations and delusions are known to increase the risk for suicide, yet these same symptoms frequently hinder proper and adequate communication with the patient [5]. Depression, also strongly associated with suicide, can easily be confused with the negative symptoms of schizophrenia, such as apathy and reduced expression, or can even be attributed to the side effects of prescribed antipsychotic regimens [1,6]. In light of these considerations, it becomes necessary to consider other factors to better predict and prevent suicide. The National Alliance for Suicide Prevention proposed the identification of peripheral biomarkers for screening and risk assessment [7]. We, in turn, adopted this approach to predict suicidal ideation specifically in the schizophrenia population.

Family studies have long supported the notion that suicidal behaviors, including both suicide attempts and completion, have a strong genetic component [8]. However, genome-wide association studies (GWAS) have been unable to consistently and reliably identify risk associated with DNA sequence variants alone. As such, in recent years, epigenetic mechanisms have been suggested to play a role in “quantifying the missing heritability” of suicide in schizophrenia [9]. DNA methylation and suicide have been well-studied in relation to the dysfunction of the adaptive stress response by the hypothalamic-pituitary-adrenal (HPA) axis, particularly in the NR3C1 and SKA2 genes [10,11,12,13,14]. Therefore, the evidence suggests that these epigenetic markers can prove valuable in investigating suicide attempts and completed suicides. However, predicting suicidal ideation may very well yield different results from those studies utilizing suicidal behaviors as the outcome variable [15]. As such, it is necessary to replicate these studies in the context of suicidal ideation. The present study will investigate genome-wide methylation status in association with suicidal ideation severity. Based on previous studies investigating DNA methylation and suicide [10,11,12,13,14], we further predict that suicide ideation will be associated with genome-wide methylation. 

## 2. Materials and Methods

### 2.1. Participants

The present study included 112 individuals with a diagnosis of schizophrenia spectrum disorders, recruited from the Centre for Addiction and Mental Health (CAMH) in Toronto, Canada. Subjects were between the ages of 18 and 75 years and did not have a past history of head trauma with loss of consciousness, nor diagnosis of an intellectual disability, major neurological disorder, or substance-induced psychosis. All participants were prescribed antipsychotics. The study was approved by the CAMH Research Ethics Board. Each subject provided consent to participate. The Columbia- Suicide Severity Rating Scale (C-SSRS) [16] was administered to determine whether subjects were experiencing suicidal ideation at the time of the assessment, and if so, the severity of ideation.

### 2.2. Sample Collection and DNA Methylation

Venous blood was collected from participants after administering the C-SSRS. Genomic DNA was extracted using the QIAamp^®^ DNA Blood Maxi Kit (QIAGEN Inc., Hilden, Germany). Samples were then sent to The Centre for Applied Genomics (TCAG) at the Hospital for Sick Children for further processing. There, 500 ng of DNA was treated with sodium bisulfite using the EZ DNA Methylation Kit (Zymo Research, Irvine, CA, USA). Genome-wide DNA methylation was quantified using the Infinium^®^ MethylationEPIC BeadChip array (Illumina) to interrogate over 850,000 CpG loci at single-nucleotide resolution. The confocal laser scanning iScan^®^ (Illumina) system was utilized to output signal intensities of the bisulfite-converted DNA methylation as IDAT files [17,18].

### 2.3. Identification of Differentially Methylated Positions and Regions

All analyses were conducted using the minfi Bioconductor package run in the R- 3.5.1 (64-bit) statistical analysis environment on the CAMH Specialized Computing Cluster. The methylation intensity data files (IDAT) and suicidal ideation severity for each corresponding subject were first analyzed. For quality control purposes, the preprocessRaw function was implemented to convert IDAT data into methylation β-values without normalization. We generated a probe intensity scatterplot and β-value density plot; samples with poor quality were excluded from further analyses [19,20]. For further downstream analyses, the preprocessFunnorm function was utilized for functional normalization to remove biological and technical variation [21]. To identify differentially methylated positions (DMPs), the dmpFinder function was used to test individual CpG sites for associations between methylation level and the suicidal ideation severity phenotype. The dmpFinder function performed univariate linear regressions of the C-SSRS score by each of the CpG positions. Considering multiple testing, a significance threshold of *p* < 5.8 × 10^−8^ was used to determine CpG sites that are significantly associated with the C-SSRS score. Differentially methylated regions (DMRs) were identified through the implementation of the bumphunter function, with a methylation differential cut-off of 0.2 corresponding to a 20% difference in *p*-values [21]. The utility of bump hunting allows for the consideration of methylation levels between nearby CpG sites, and hence the identification of regions that are differentially methylated [17,22]. For DMR, the *p*-value was used to determine the significance of the association between candidate bumps and the C-SSRS score. For all analyses, only autosomal positions and regions were included and no other probe filtering were applied [23]. Cell composition was estimated using the Horvath algorithm (https://horvath.genetics.ucla.edu/html/dnamage (accessed on 31 March 2022)). 

## 3. Results

### 3.1. Demographic and Clinical Characteristics

Among our cohort of 112 participants, we found that 19 subjects, or approximately 16.8%, currently reported experiencing suicidal ideation, while the remaining 94 subjects did not report suicidal ideation. The demographic and clinical variables from these participants are summarized in Table 1. Consistent with previous findings, we identified significant group differences between ideators and non-ideators with respect to psychosis severity, depression, hopelessness, and perceived stress [1,5,24,25].

### 3.2. Quality Control Assessments

**Figure 1:**
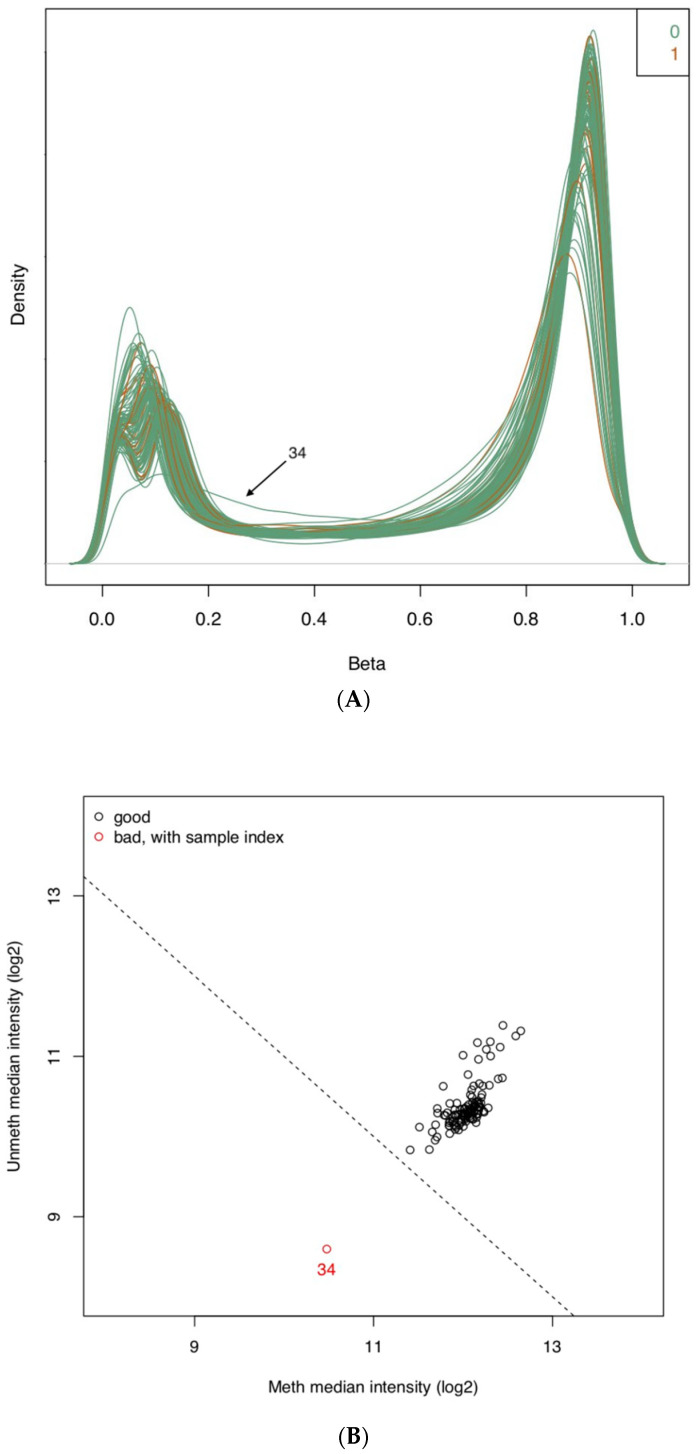
(**A**): *β*-value density plot for quality control purposes. Each line represents the density distribution of methylation levels for each CpG site throughout the genome. The number 0 and associated green lines represent samples from subjects not reporting suicidal ideation, whereas the number 1 and corresponding red lines represent samples from subjects reporting current suicidal ideation. The two peaks in the distribution curve are seen near 0.0 and 1.0 and refer to the theoretical states of CpG sites being completely methylated or unmethylated. (**B**): Probe intensity scatterplot for quality control purposes. The logarithm of median methylated and unmethylated signal intensities was plotted. High quality appears to cluster with high signal intensities (black), whereas low-quality samples are located separately from the main cluster with lower signal intensities (red). One sample with bad quality was identified and indicated in red, along with the sample index number. The sample index number allows for the identification of a given sample for removal in subsequent analyses.

The probe intensity scatterplot graphically represents the logarithm of the median intensity of the methylated signal against the logarithm of the median intensity of the unmethylated signal (Figure 1A). Typically, high-quality samples cluster together with high median methylated and unmethylated intensities, whereas low-quality samples are located separately from the main cluster at lower medians [26]. Our results indicated that one sample was considered to be an outlier based on the above definitions. Following the conversion of raw IDAT data into methylation levels, we then generated a β-value density plot (Figure 1B), a visual representation that allows for the identification of sample outliers with poor quality. We observed that there was an overall level of consistency in the density plot, though one sample showed relatively poor quality. This was confirmed to be the same subject as identified with the probe intensity scatterplot and was removed from further analyses. The QC analysis confirms the findings illustrated in Figure 1A,B.

### 3.3. Differentially Methylated Positions (DMPs)

**Figure 2:**
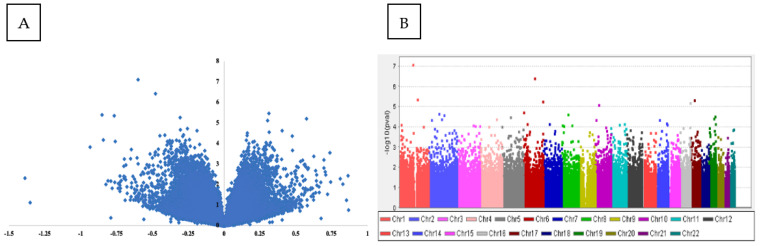
(**A**): Volcano plot of DMPs associated with suicidal ideation severity, a scatterplot representation of the association *p*-values for all CpG sites; the x-axis indicates the beta coefficient. The y-axis represents the effect size. (**B**): Manhattan plot of DMPs associated with suicidal ideation severity. Scatterplot representation of the association *p*-values for all CpG sites across the genome, arranged in order based on chromosome and position. The *y*-axis represents the negative logarithm of *p*-values.

The dmpFinder function was utilized to identify DMPs between subjects with and without suicidal ideation, considering their suicidal ideation severity scores. As defined by the C-SSRS, this continuous phenotype ranged from a score of zero to five, with increasing severity. The ten most significant CpG sites that were identified using this approach are reported in Table 2. Using LIMMA, we further analyzed the top ten CpG sites using a regression model that incorporated age, sex, ethnicity, and plate and cell composition (granulocytes, lymphocytes, and monocytes) as covariates. Only two of the top ten CpG sites remained significant after the addition of covariates (Table 2). The association *p*-values for all CpG sites across the genome before correction are graphically represented with volcano and Manhattan plots (Figure 2). In the original analysis, we determined that cg27077219, located within the LINC01356 gene on chromosome 1, was hypomethylated in subjects experiencing current suicidal ideation. However, after covariate correction, this CpG site was no longer significant.

### 3.4. Differentially Methylated Regions (DMRs)

In the investigation of DMRs with *bumphunter*, we identified a total of 575 regions that were differentially methylated and nominally associated with current suicidal ideation. A list of the ten most significant DMRs are shown in Table 3. The methylation difference value represents the percent difference in methylation at a particular DMR between subjects with and without suicidal ideation. Positive methylation differences were indicative of a particular region being hypermethylated, and negative differences indicated hypomethylation of the DMR in subjects with current suicidal ideation. We report that a DMR located in chromosome 10 with a start position at 79655482 was hypermethylated in subjects with current suicidal ideation (top hit).

## 4. Discussion

In the present study, we assessed differential DNA methylation across the genome at the level of individual positions (DMPs) and regions (DMRs). To the best of our knowledge, this study is the first to investigate genome-wide methylation in relation to suicidal ideation severity in schizophrenia. We identified several DMPs and DMRs associated with suicidal ideation severity, although there was no consistency between DMR and DMP analyses and we were unable to conclude that these findings were significant at the genome-wide level. When applying a Bonferroni correction for 850,000 CpG sites, as in the DMP test, the genome-wide significance would require a *p* < 5.8 × 10^−8^. For the DMRs, the threshold would be less stringent. Considering that the human genome has approximately 30,000 CpG islands [27], the genome-wide significance would require a *p* < 1.6 × 10^−6^. Our top DMPs and DMRs were therefore only found to have suggestive levels of significance at the genome-wide level since they were not corrected for multiple tests.

Previous studies on DNA methylation and suicidal behavior have specifically pointed to hypomethylation of the NR3C1 gene [10]. However, we were unable to find similar results in our analysis of DNA methylation and current suicide ideation severity. Moreover, the CpG sites highlighted in our study also did not coincide with a previously published genome-wide methylation study on suicide attempts [9]. Additionally, a recent genome-wide methylation analysis showed hypomethylation of the PSORS1C3 in suicide victims, although we were unable to find similar results [28].

Despite these results, our study had several strengths. From a technical standpoint, we utilized the latest Infinium^®^ MethylationEPIC BeadChip array with the most comprehensive coverage of over 850,000 CpG sites, compared to previous generations of methylation arrays which covered either only 450,000 or 27,000 sites. The MethylationEPIC array removed approximately 10% of CpG sites found in the 450K chip due to poor performance, and among others, added 333,265 CpG sites located on intergenic and gene enhancer regions [29,30]. Prior to the identification of DMPs and DMRs, we conducted an array of quality control assessments to identify and remove samples of poor quality, as well as extra steps to process and normalize methylation measures.

Furthermore, our study design considered both differentially methylated positions (DMPs) and regions (DMRs) in an annotation-free approach. We were not the first to utilize these methods, with another study identifying both DMPs and DMRs associated with psychotic experiences [31]. Nonetheless, it is a strength of this study. In fact, it was even suggested that both approaches be run in tandem, since individual DMPs are not necessarily evenly spaced across the genome, and in many cases are not located within 1 kbp of a neighboring site [32]. While the identification of DMPs is certainly of interest, the region analysis is generally considered more robust than individual probes. Bump hunting for regions that are differentially methylated is more likely to identify differentially expressed genes than probing for individual CpG sites within the genome [19]. The identified genes would then have the potential to lead to the identification of downstream associated pathways and a greater understanding of etiological factors contributing to suicidal ideation.

Several limitations are present in this study. While our study had a reasonable size, a larger sample would have been indicated for improved interpretation of genome-wide results [33]. Furthermore, in the investigation of psychotic experiences mentioned above, the authors utilized a sample size of 845 participants [31]. Further studies with increased sample sizes are thus required. Additionally, the present study employs a cross-sectional design to investigate DNA methylation in relation to the severity of suicidal ideation; however, the causal impact of DNA methylation should be investigated longitudinally in the future so that additional factors such as financial, health, and family stress can be analyzed. Probes that are directly affected by SNPs, such as those associated with suicidality or schizophrenia, should be included in further investigations. Finally, future studies should examine methylation change in post-mortem neural tissue, especially considering the fact that genetic liability of schizophrenia has long been emphasized, with gray matter reductions in the anterior cingulate being reported as a marker of genetic liability for psychosis [34]. Although previous literature demonstrates that there is a limited, albeit significant degree of correspondence between peripheral blood methylation and brain methylation, causal effects should be interpreted as tentative since CpG sites may differ by tissue type.

## 5. Conclusions

We investigated individual sites and regions across the genome that were differentially methylated in association with current suicidal ideation severity. While the present hypothesis-free study did not determine any positions or regions differentially methylated that were significant at the genome-wide level, our findings suggest trends toward significance. While epigenome-wide association studies are still in their infancy, further work is required to replicate, support, and validate our findings presented here. The present analysis can be further expanded to determine the clinical utility of using genome-wide methylation markers to determine current suicide ideation severity.

In conclusion, despite the lack of statistical power to discover genome-wide changes for biomarker identification, this study might stimulate further investigation addressing the biological relevance of genome-wide methylation in psychiatric disorders.

## Figures and Tables

**Table 1:** Demographic and clinical variables in the study cohort presented as mean ± standard deviation. Variables were also tested for group differences between subjects with and without current suicidal ideation with the Mann-Whitney U test for continuous variables and the Chi-square test for categorical variables.

Total (*N* = 112)	Ideator (*n* = 19)	Non-Ideator (*n* = 93)	*p*-Value
Sex (male/female)	11/8	59/34	0.377
Age (years)	44.7 ± 9.3	44.9 ± 12.8	0.961
Age-of-Onset (years)	21.2 ± 6.1	22.9 ± 6.8	0.345
Duration-of-Illness (years)	23.1 ± 9.9	21.5 ± 13.3	0.503
BPRS (Brief Psychiatric Rating Scale)	33.2 ± 7.8	28.3 ± 6.6	0.013
CDSS (Calgary Depression Scale for Schizophrenia)	6.7 ± 5.3	2.8 ± 3.2	0.031
BHS (Beck Hopelessness Scale)	7.6 ± 6.8	4.1 ± 4.3	0.043
PSS (Perceived Stress Scale)	31.2 ± 6.6	24.1 ± 6.8	0.012
SAI (Schedule for Assessment of Insight)	11.1 ± 2.6	11.6 ± 3.1	0.707

**Table 2:** List of top ten differentially methylated positions (DMPs) associated with current suicidal ideation severity. Methylation at these CpG sites were identified to be associated with suicidal ideation severity, as a continuous phenotype, with a linear regression-based algorithm.

Chr	Position	CpG Site	Gene	*p*-Value	*β* Coefficient	*p*-Value after Correction *
1	113392580	cg27077219	*LINC01356*	7.85 × 10^−8^	−0.600	0.247351
6	97285662	cg14723344	*GPR63*	3.85 × 10^−7^	−0.478	0.015739
1	153044071	cg13950674	*SPRR2B*	4.08 × 10^−6^	−0.851	9.9181 × 10^−7^
17	33772796	cg00888402	*SLFN13*	4.54 × 10^−6^	−0.766	0.000008
6	168629778	cg01801443	Intergenic	5.47 × 10^−6^	−0.306	0.000003
16	89299756	cg27334271	Intergenic	6.39 × 10^−6^	0.574	0.000029
10	30692613	cg02903852	Intergenic	7.65 × 10^−6^	0.302	0.001251
6	5951562	cg12116564	Intergenic	1.92 × 10^−5^	−0.257	6.2654 × 10^−7^
2	88355002	cg06459916	*KRCC1*	2.20 × 10^−5^	−0.305	0.178508
8	55380008	cg17993900	Intergenic	2.37 × 10^−5^	−0.188	0.005229

Chr = chromosome number; Position = base-pair coordinate of the CpG site; *p*-Value = significance of the differentially methylated position associated with current suicidal ideation severity; *β* Coefficient = regression coefficient. * Results corrected for age, sex, ethnicity, batch, monocyte count, lymphocyte count and granulocyte count.

**Table 3:** List of top ten differentially methylated regions (DMRs) associated with current suicidal ideation. These regions were identified to be the most differentially methylated between subjects with and without current suicidal ideation.

Chr	Position	Gene	Methylation Difference (%)	*p*-Value
10	79655482	*DLG5*	27.78	1.04 × 10^−3^
1	2100232	*CACNB4*	27.65	1.14 × 10^−3^
12	49074303	*KANSL2*	−26.56	1.66 × 10^−3^
22	43168851	Intergenic	−23.77	4.89 × 10^−3^
11	118022607	*SCN4B*	−23.54	5.41 × 10^−3^
13	103423502	*TEX30*	−23.53	5.41 × 10^−3^
9	135937572	*CEL*	−22.90	7.59 × 10^−3^
1	152572665	*LCE3C*	−15.32	7.80 × 10^−3^
12	123757860	*CDK2AP1*	21.95	9.88 × 10^−3^
1	152586240	*LCE3B*	−21.63	1.10 × 10^−2^

Chr = chromosome number; Position = base-pair coordinate of the beginning of the DMR; Methylation Difference = difference in the methylation levels (%) between subjects with and without suicidal ideation; *p*-Value = significance of the differentially methylated region associated with current suicidal ideation. Nominal *p*-value presented.

## Data Availability

Data available on request.
